# CNDP1 (CTG)_5_ allele and cardiovascular events in high-risk patients: LURIC study results

**DOI:** 10.1038/s41598-026-49233-4

**Published:** 2026-04-21

**Authors:** Steffen A. Hettler, Angela Moissl, Graciela E. Delgado, Heyko Skladny, Winfried März, Bernhard K. Krämer, Benito A. Yard, Marcus E. Kleber

**Affiliations:** 1 Vth Department of Medicine (Nephrology, Hypertensiology, Rheumatology, Endocrinology, Pneumology), Medical Faculty Mannheim, University Medical Center Mannheim, University of Heidelberg, Mannheim, Germany; 2 Department of Hematology and Oncology, Children’s University Hospital Tübingen, University of Tübingen, Tübingen, Germany; 3 Synlab MVZ Humangenetik Mannheim, Mannheim, Germany; 4 Synlab Academy, Synlab Holding Deutschland GmbH, Augsburg/Mannheim, Germany

**Keywords:** Carnosine, CNDP1, Cardiovascular mortality, Diabetes mellitus, Diabetic kidney disease, LURIC, Chronic kidney disease, Cardiovascular genetics, Disease genetics, Risk factors, Diabetes complications, Genetics research

## Abstract

Homozygous carriers of the CNDP1 (CTG)_5_ allele with diabetes mellitus are believed to have a lower risk of developing diabetic kidney disease compared to individuals carrying alleles with higher numbers of this CTG repeat. However, recent studies claimed that homozygosity for the (CTG)_5_ allele increases the risk of disease progression towards end-stage renal disease and even cardiovascular mortality, at least in women. Therefore, this study sought to confirm in a prospective manner in a cardiovascular high-risk cohort if individuals with two (CTG)_5_ alleles indeed have an increased cardiovascular mortality. 3,201 individuals from the LURIC study were included and followed for a median of 9.9 years. CNDP1 (CTG)_n_ genotypes were assessed and related to all-cause and cardiovascular mortality. 1,157 (36.1%) patients carried the homozygous CNDP1 (CTG)_5_ genotype. No significant difference for all-cause and cardiovascular mortality was detected after multiple adjustments for cardiovascular risk factors, neither for the whole cohort nor for men or women, respectively. In this prospective cardiovascular high-risk cohort, homozygosity for the CNDP1 (CTG)_5_ allele was not associated with increased cardiovascular mortality compared to all other genotypes together. These findings do not confirm previous reports suggesting a sex-specific increase in cardiovascular mortality among women carrying two (CTG)_5_ alleles.

**Supplementary Information:**

The online version contains supplementary material available at 10.1038/s41598-026-49233-4.

## Introduction

Diabetes mellitus (DM) is one of the most common chronic diseases world-wide with steadily increasing yearly prevalence in adults^1^. As a consequence, also the incidence of diabetic complications, e.g. diabetic kidney disease (DKD), is increasing. DKD is a serious complication in patients with DM and the leading cause for end-stage renal disease (ESRD)^2^ requiring renal replacement therapy. Development and progression of DKD is influenced by a number of modifiable (e.g. glycemic control, hypertension and dyslipidemia)^3–6^ and non-modifiable risk factors (e.g. sex and genetic profile)^7,8^. There is ample evidence that DKD underlies genetic traits and a number of susceptibility loci have been suggested. As such, different polymorphisms in the carnosinase 1 (CNDP1) promoter and gene were found to be associated with DKD. These include a single nucleotide polymorphism (SNP) rs2346061^9,10^ and a trinucleotide repeat polymorphism D18S880 in exon 2. The later encodes a leucine (CTG) repeat (in Caucasians 4–7 repeats)^11,12^ in the signal peptide of CNDP1 and is believed to be relevant for its secretion^13^. CNDP1 is primarily expressed in brain and liver from where it is secreted in cerebrospinal fluid and plasma respectively. CNDP1 hydrolyzes histidine containing dipeptides, i.e. carnosine, homo-carnosine and anserine^14,15^. The former dipeptide may act as an antioxidant^16^, lowers plasma glucose^17,18^ and inhibits protein carbonylation and glycoxidation^19,20^. Considering these biochemical properties, it appears plausible that carnosine might attenuate hyperglycemia-induced damage and that polymorphisms of CNDP1 resulting in an altered carnosine metabolism might influence the course of DKD (The key results of the studies mentioned below can be found in Supplementary Table 1.): While a low number of CTG repeats in CNDP1 is associated with lower plasma CNDP1 concentrations it seems that the proportion of patients with type 2 DM and DKD is higher amongst individuals that carry higher numbers of CTG repeats^11,13,21^. Indeed, it was shown that homozygous carriers of a short allelic form, i.e. (CTG)_5_, had a lower risk to develop DKD^11,12^. Other studies have corroborated the early findings and suggested that the renoprotective effect afforded by the (CTG)_5_ allele might be restricted to certain ethnicities like Caucasians and people from North India or Malaysia^9,10,22,23^, the type of DM^24^ and sex^21,25^. A prospective study in patients with type 1 DM suggested that the (CTG)_n_ polymorphism predicts progression to ESRD in patients with DKD^26^. The authors of the later study postulated that patients with type 1 DM and DKD are at increased risk of progressing to ESRD when carrying two (CTG)_5_ alleles^26^. This prompts the question if the beneficial findings from the previously mentioned cross-sectional studies^9–12,22,25^ might also be explained by a survival disadvantage of the (CTG)_5_ allele in view of the fact that patients with DKD have a high risk for cardiovascular complications^27^. In fact, in another prospective study with 871 type 2 DM patients (ZODIAC cohort) the same group could detect an increased risk for cardiovascular mortality in women with two (CTG)_5_ alleles^28^. Since these findings are in contrast to the physiological properties of the carnosine-carnosinase system, this study sought to prospectively assess if there is a sex-specific association between homozygosity of the (CTG)_5_ allele and increased cardiovascular mortality in a cardiovascular high-risk cohort.

## Methods and materials

### Study cohort

The methodology and baseline characteristics of the Ludwigshafen Risk and Cardiovascular Health (LURIC) study were previously reported in detail^29^. Briefly, the LURIC study is a prospective, exploratory ongoing study to investigate environmental and genetic risk factors of cardiovascular diseases, such as coronary artery disease (CAD) and type 2 DM. 3,316 individuals of German ancestry (2,310 men and 1,006 women) who mostly underwent coronary angiography were included between 1997 and 2000. The study was approved by the ethics committee of the Landesärztekammer Rheinland-Pfalz and was conducted according to the Declaration of Helsinki. Informed written consent was obtained from all participants.

### Laboratory measurements

Fasting venous blood samples were collected under standardized conditions when entering the study. The analytic methods of selected biomarkers involved in the pathogenesis of cardiovascular complications such as N-terminal pro-B-type natriuretic peptide (NT-pro-BNP), high-sensitivity C-reactive protein (hsCRP), interleukin-6 (IL-6), triglycerides, low-density lipoprotein (LDL) cholesterol, high-density lipoprotein (HDL) cholesterol, fasting plasma glucose, glycated hemoglobin (HbA_1c_) and creatinine have been described earlier^29^. The estimated glomerular filtration rate (eGFR) was calculated using the serum creatinine and cystatin C based CKD-EPI equation^30^.

### Definition of clinical variables

Coronary artery disease (CAD) was examined by angiographic criteria using the maximum luminal narrowing. The three major coronary arteries were divided into 15 segments according to the American Heart Association^31^. CAD was defined as the presence of visible lumen narrowing (> 20% stenosis) in at least one segment.

Diabetes mellitus was defined according to the guidelines of the American Diabetes Association from 2010^32^.

Blood pressure was measured repeatedly (at least 3 times) with an automated oscillometric device while supine for at least 10 min^29^. The average was obtained from the last two measures of systolic and diastolic blood pressure. Hypertension was diagnosed if blood pressure was ≥ 140 (systolic) and/or ≥ 90 mmHg (diastolic) according to the ESC/ESH guidelines from 2018^33^ or if patients were on antihypertensive medication.

Smoking status was retrieved using questionnaires and verified by measurement of serum cotinine concentration (active smokers vs. non- or ex-smokers)^34^. The cut-off for defining active smoking was 15 µg/l as described earlier^35,36^.

Definition of macrovascular complications was made similar to^28^: History of CAD or peripheral artery occlusive disease or carotid stenosis or stroke.

### Genotyping of the CTG repeat polymorphism

Genomic DNA was isolated from white blood cells in several laboratories collaborating in the LURIC project using commercially available DNA extraction kits or their own salting out methods^29^. DNA was then stored at −20°C for later use. Genotyping was performed using standard PCR protocols using a fluorescence-labeled forward primer (5’FAM-GTTCCTCCCATGTCAAACCCTTCC-3’) and an unlabeled reverse primer (5’-TACCTGCACAAATTCATCCTGATGG-3’). The annealing temperature was 57 °C. After PCR, amplification genotyping by means of fragment analysis was performed on a 3730xl DNA Analyzer (Applied Biosystems) to determine the number of trinucleotide repeats of exon 2 in each allele. Automatic genotyping was performed using GeneMapper (version 3.7, Applied Biosystems). Three alleles were observed with fragment sizes of 157, 160 and 163 base pairs corresponding to five, six and seven leucine repeats, respectively.

### Follow-up assessments and definition of end-points

Participants were followed up for a median of 9.9 years. Information on vital status was obtained from local community registries. Death certificates were reviewed by two blinded, experienced clinicians to classify the deceased into those who died from cardiovascular and non-cardiovascular events. Death from cardiovascular causes included sudden cardiac death, fatal myocardial infarction, death due to heart failure, death after intervention to treat CAD, stroke and other deaths due to heart diseases. In cases of disagreement or uncertainty concerning the cause of death, the decision was made by a principal investigator.

### Statistical analysis

Normally distributed continuous variables were reported as the mean ± standard deviation (SD), while variables with skewed distribution were reported as medians with interquartile ranges (IQR). Categorical data were presented as percentages.

For normally distributed variables comparisons between groups were performed using unpaired Student’s t-tests, whereas for non-normally distributed variables Wilcoxon-Mann-Whitney tests were used. Chi-square tests were used to compare categorical variables.

Different Cox proportional hazards survival regression models were applied to analyze the influence of the (CTG)_5_ allele on all-cause and cardiovascular mortality (Table 1).

**Table 1:** Summary of applied cox proportional hazards models.

Model	Inclusion criteria	Adjustment	Outcome
1	-	-	• All-cause mortality • Cardiovascular mortality
2	-	Age, sex	• All-cause mortality • Cardiovascular mortality
3	Patients with diabetes mellitus	Age, sex	• All-cause mortality • Cardiovascular mortality
4	-	Age, sex, BMI, smoking status, history of macrovascular complications, systolic blood pressure, HbA_1c_, total cholesterol/HDL ratio, plasma creatinine	• All-cause mortality • Cardiovascular mortality
5	Patients with diabetes mellitus	Age, sex, BMI, smoking status, history of macrovascular complications, systolic blood pressure, HbA_1c_, total cholesterol/HDL ratio, plasma creatinine	• All-cause mortality • Cardiovascular mortality

Abbreviations: *BMI* body mass index, *HbA*_*1c*_ glycated hemoglobin, *HDL* high density lipoprotein.

Model 1 (crude model), model 2 (adjusted for age and sex), model 3 (only patients with diabetes mellitus, adjusted for age and sex), model 4 (adjusted for age, sex, body mass index, smoking status, history of macrovascular complications, systolic blood pressure, HbA_1c_, total cholesterol/HDL ratio, plasma creatinine) and model 5 (only patients with diabetes mellitus, adjusted for age, sex, body mass index, smoking status, history of macrovascular complications, systolic blood pressure, HbA_1c_, total cholesterol/HDL ratio, plasma creatinine). Model 5 mimics the model where the increased cardiovascular mortality of women being homozygous carriers of the (CTG)_5_ allele was observed^28^ except for the urinary albumin excretion since urinary samples were not obtained in the LURIC study and diabetes duration since data was not available for all patients.

All tests were two-sided and a p-value of < 0.05 was considered statistically significant. All analyses were carried out using SPSS (version 27.0.0, IBM) and R (version 4.3.1, R Foundation for Statistical Computing)^37^.

## Results

### Patient characteristics at baseline

Data on CNDP1 genotype was available for 3,201 participants. 1,157 patients (36.1%) were homozygous carriers of the CNDP1 (CTG)_5_ allele. The frequency of CNDP1 (CTG)_n_ genotypes is shown in Table 2.

**Table 2:** Frequency of CNDP1 (CTG)_n_ genotypes.

CNDP1 (CTG)_*n*_ genotype	5/5 homozygous	Others					Total
		5/6 heterozygous	5/7 heterozygous	6/6 homozygous	6/7 heterozygous	7/7 homozygous	
Frequency	1,157 (36.1%)	1,331 (41.6%)	185 (5.8%)	426 (13.3%)	97 (3.0%)	5 (0.2%)	3,201 (100%)

Data are presented as absolute numbers and percentages.

Baseline characteristics of the study population are shown in Table 3. No significant differences were found between the homozygous CNDP1 (CTG)_5_ and all other CNDP1 (CTG)_n_ genotypes together (referred as “others”) except for a slightly higher number of patients with hypertension in the latter group.

**Table 3:** Baseline characteristics in relation to CNDP1 (CTG)_n_ genotypes.

Variable	Homozygous (CTG)_5_	Others	*p*
Demographic characteristics			
Age (years)	62.8 ± 10.7	62.4 ± 10.5	0.347
Female sex (%)	30.4	30.2	0.907
**Clinical characteristics**			
BMI (kg/m^2^)	27.5 ± 4.10	27.6 ± 4.08	0.570
BP systolic (mmHg)	141 ± 23.8	141 ± 23.5	0.749
BP diastolic (mmHg)	80.9 ± 11.4	81.1 ± 11.7	0.711
**Laboratory parameters**			
LDL-Cholesterol (mg/dl)	117 ± 35.2	116 ± 32.9	0.469
HDL-Cholesterol (mg/dl)	38.8 ± 10.6	38.5 ± 11.1	0.577
Triglycerides (mg/dl)	145 (110–201)	149 (109–201)	0.619
HbA_1c_ (%)	6.32 ± 1.25	6.31 ± 1.25	0.804
hsCRP (mg/l)	3.35 (1.26–8.71)	3.44 (1.37–8.44)	0.560
IL-6 (ng/l)	3.19 (1.77–6.08)	3.16 (1.81–6.08)	0.865
NT-pro-BNP (pg/ml)	281 (101–837)	308 (117–917)	0.086
eGFR (ml/min/1.73m^2^)	82.0 ± 20.0	81.3 ± 20.5	0.352
**Comorbidities**			
Coronary artery disease (%)	77.1	78.8	0.266
Macrovascular complications (%)	79.1	80.8	0.265
Diabetes mellitus (%)	40.0	39.7	0.876
Hypertension (%)	71.5	75.1	0.032
Active smokers (%)	23.4	23.9	0.755

Data are presented as the mean ± SD or the median (interquartile range) or proportion.

For normally distributed variables comparisons between groups were performed using unpaired Student’s t-tests, whereas for non-normally distributed variables Wilcoxon-Mann-Whitney tests were used. Chi-square tests were used to compare categorical variables.

Abbreviations: *BMI* body mass index, *BNP* brain natriuretic peptide, *BP* blood pressure, *eGFR* estimated glomerular filtration rate, *HbA*_*1c*_ glycated hemoglobin, *HDL* high density lipoprotein, *hsCRP* high sensitive C-reactive protein, *IL-6* interleukin-6, *LDL* low density lipoprotein.

### Association of the CNDP1 (CTG)_5_ homozygous genotype with mortality

**Fig. 1:**
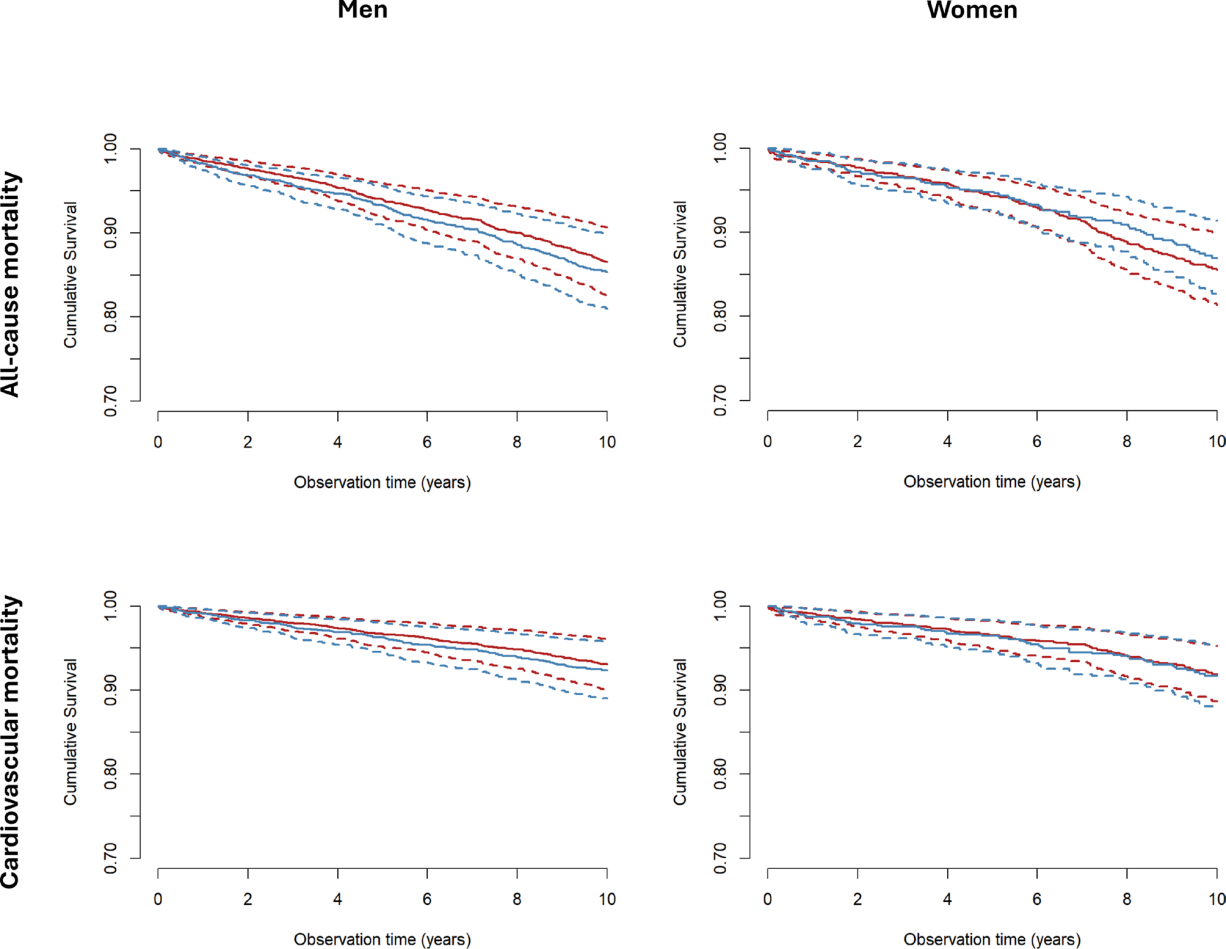
Sex-dependent cumulative survival of all patients with diabetes mellitus. All-cause (upper row) and cardiovascular (lower row) mortality are depicted according to model 5 for carriers of the homozygous CNDP1 (CTG)_5_ (blue) vs. all other (red) genotypes. Survival estimates are depicted as solid lines, with 95% confidence intervals shown as dashed lines.

During follow-up, 962 patients died (30.1% of the study cohort), of which 600 died due to cardiovascular events. Table 4 shows hazard ratios for all-cause and cardiovascular mortality in five Cox regression models for men, women or both together either or not adjusted for different confounders. Irrespective of the applied model, no significant difference was found between the groups. Survival curves of men and women with diabetes mellitus for all-cause and cardiovascular mortality adjusted for established cardiovascular risk factors (model 5) are displayed in Fig. 1.

**Table 4:** Hazards ratios (95% CI) for all-cause and cardiovascular mortality according to CNDP1 genotype for five different Cox regression models.

	Total *n* = 3,201	Men *n* = 2,230	Women *n* = 971
**Model 1 (no adjustment)**			
*All-cause mortality*			
Others	1.0 (reference)	1.0 (reference)	1.0 (reference)
Homozygous (CTG)_5_	1.00 (0.876–1.140)	1.02 (0.873–1.190)	0.95 (0.735–1.230)
	*p* = 0.996	*p* = 0.817	*p* = 0.695
*Cardiovascular mortality*			
Others	1.0 (reference)	1.0 (reference)	1.0 (reference)
Homozygous (CTG)_5_	1.06 (0.899–1.250)	1.04 (0.856–1.260)	1.12 (0.817–1.530)
	*p* = 0.486	*p* = 0.698	*p* = 0.482
**Model 2 (adjustment for age and sex)**			
	Total *n* = 3,201	Men *n* = 2,230	Women *n* = 971
*All-cause mortality*			
Others	1.0 (reference)	1.0 (reference)	1.0 (reference)
Homozygous (CTG)_5_	1.04 (0.912–1.190)	1.06 (0.910–1.240)	0.98 (0.757–1.260)
	*p* = 0.558	*p* = 0.450	*p* = 0.861
*Cardiovascular mortality*			
Others	1.0 (reference)	1.0 (reference)	1.0 (reference)
Homozygous (CTG)_5_	1.10 (0.935–1.300.935.300)	1.08 (0.890–1.310)	1.15 (0.840–1.580)
	*p* = 0.246	*p* = 0.433	*p* = 0.379
**Model 3 (only patients with diabetes mellitus**,** adjustment for age and sex)**			
	Total *n* = 1,277	Men *n* = 888	Women *n* = 389
*All-cause mortality*			
Others	1.0 (reference)	1.0 (reference)	1.0 (reference)
Homozygous (CTG)_5_	0.96 (0.805–1.150)	0.98 (0.799–1.210)	0.90 (0.638–1.280)
	*p* = 0.654	*p* = 0.861	*p* = 0.570
*Cardiovascular mortality*			
Others	1.0 (reference)	1.0 (reference)	1.0 (reference)
Homozygous (CTG)_5_	1.02 (0.824–1.270)	1.00 (0.779–1.300.779.300)	1.08 (0.720–1.620)
	*p* = 0.843	*p* = 0.972	*p* = 0.711
**Model 4 (adjustment for age**,** sex and other confounders**^**1**^**)**			
	Total *n* = 3,189	Men *n* = 2,220	Women *n* = 969
*All-cause mortality*			
Others	1.0 (reference)	1.0 (reference)	1.0 (reference)
Homozygous (CTG)_5_	1.05 (0.922–1.200.922.200)	1.10 (0.939–1.280)	0.95 (0.730–1.220)
	*p* = 0.448	*p* = 0.243	*p* = 0.670
*Cardiovascular mortality*			
Others	1.0 (reference)	1.0 (reference)	1.0 (reference)
Homozygous (CTG)_5_	1.10 (0.930–1.300)	1.11 (0.909–1.340)	1.10 (0.797–1.510)
	*p* = 0.269	*p* = 0.314	*p* = 0.571
**Model 5 (only patients with diabetes mellitus**,** adjustment for age**,** sex and other confounders**^**1**^**)**			
	Total *n* = 1,272	Men *n* = 883	Women *n* = 389
*All-cause mortality*			
Others	1.0 (reference)	1.0 (reference)	1.0 (reference)
Homozygous (CTG)_5_	0.962 (0.806–1.150)	0.986 (0.800–1.210)	0.90 (0.630–1.290)
	*p* = 0.673	*p* = 0.893	*p* = 0.573
*Cardiovascular mortality*			
Others	1.0 (reference)	1.0 (reference)	1.0 (reference)
Homozygous (CTG)_5_	1.01 (0.815–1.260)	1.01 (0.780–1.310)	1.11 (0.730–1.690)
	*p* = 0.913	*p* = 0.942	*p* = 0.623

^1^: body mass index, smoking status, history of macrovascular complications, systolic blood pressure, HbA_1c_, total cholesterol/HDL ratio, plasma creatinine.

## Discussion

Irrespective of DM, our study did not corroborate the findings of an increased risk for cardiovascular mortality in homozygous carriers of the CNDP1 (CTG)_5_ allele^28^, neither for the whole cohort, nor specifically for women.

The study of Alkhalaf et al.^28^ is in a number of aspects well comparable with the present one. Firstly, the vast majority of participants in both cohorts are Caucasian which is quite important since the protective properties of the CNDP1 (CTG)_5_ polymorphism seem to depend on the genetic background^9,10,22,23^. Secondly, in both cohorts (ZODIAC and LURIC) the percentage of homozygous CNDP1 (CTG)_5_ individuals was almost identical (37.9% vs. 36.1% respectively)^28^, arguing that there was no difference in genotype distribution at study begin that could be attributed to a different cardiovascular risk profile in both cohorts. Thirdly, patient follow-up in both cohorts were 9.5^28^ vs. 9.9 years in the LURIC cohort, thus ruling out that the absence of an increased cardiovascular mortality risk in the LURIC cohort could underly an insufficiently long follow-up.

At second glance there are differences in the models applied to analyze the influence of the (CTG)_5_ allele on all-cause and cardiovascular mortality as well as in the study cohort that might partially explain the different results of both studies. On the one hand no urine samples were collected in the LURIC study. Therefore, albuminuria couldn’t be taken into consideration for the adjustment. Since albuminuria is an independent risk factor for cardiovascular diseases (also independent of the glomerular filtration rate that was comparable between both cohorts) it cannot be completely ruled out that this might be the cause for the different results of both studies^38–41^.

Moreover, the cardiovascular risk profile of both study cohorts differs substantially. While the study of Alkhalaf et al.^28^ was conducted to test different forms of assisted primary care in 871 patients with type 2 DM, with DM being therefore the leading cardiovascular risk factor in that cohort^42^, the inclusion criteria for the LURIC study were acute coronary syndrome (i.e. established cardiovascular disease) and indication for coronary angiography^29^. Also, the number of patients included was higher, i.e. 3,201 patients of which 1,277 suffering from DM were included. Considering the younger age in the LURIC cohort (62.7 vs. 68.0 years) this may indicate that these patients might have a lower total cardiovascular risk although the cardiovascular mortality in both studies were comparable (17% vs. 19.5% in the LURIC study for homozygous CNDP1 (CTG)_5_ and 15% vs. 18.5% in the LURIC study for all other genotypes respectively)^28^. However, taking into consideration the much higher all-cause mortality in the ZODIAC cohort (41% vs. 29.9% for homozygous CNDP1 (CTG)_5_ and 38% vs. 30.1% for all other genotypes respectively) and the vague definition of cardiovascular death in that study^43,44^, this might indicate that not all cardiovascular deaths were assessed appropriately. Therefore, the real cardiovascular mortality might have been significantly higher in the study of Alkhalaf et al. compared to the LURIC study. If the homozygous CNDP1 (CTG)_5_ genotype indeed increases cardiovascular mortality, that could possibly explain why the higher mortality was not detectable in the LURIC study when presuming a lower total cardiovascular risk in this cohort.

Thirdly, HbA_1c_ was significantly lower in the LURIC study (6.31% vs. 7.35%). Since hyperglycemia enhances carnosinase secretion and activity vice versa^45,46^ influence of the homozygous CNDP1 (CTG)_5_ allele might be more evident in cohorts with poor glycemic control like the previous one.

In summary this prospective study demonstrates that women being homozygous for the CNDP1 (CTG)_5_ allele do not have an increased cardiovascular risk and therefore disproves a previous study where such an effect was found^28^. Both studies partially differ in their cardiovascular risk profiles (age, percentage of patients with diabetes mellitus and cardiovascular disease, HbA_1c_) but importantly with the exception of HbA_1c_ the allelic variability of the carnosinase did not potentially influence these baseline characteristics^12,47^. Future phenome-wide association study analyses could provide additional insights into the broader phenotypic associations of this variant.

## Supplementary Information

Below is the link to the electronic supplementary material.

Supplementary Material 1

## Data Availability

The data underlying this article are sensitive health data and cannot be shared publicly due to privacy reasons. The data will be shared on reasonable request to the corresponding author.
