# Supplementary material for: CNDP1 (CTG)5 allele and cardiovascular events in high-risk patients: LURIC study results
Source: Sci Rep. 2026 Apr 21;16:13011. doi: 10.1038/s41598-026-49233-4 (PMC13100134; doi:10.1038/s41598-026-49233-4)
Supplement: Supplementary file 1 — Supplementary Material 1 [file 41598_2026_49233_MOESM1_ESM.docx]

**Table S1: Reported Effects of the CNDP1 (CTG)_5_ Polymorphism**

| **Year of publication** | **Key finding** | **Ethnicities included** | **Effect size** | **p-Value** | **Reference** |
| --- | --- | --- | --- | --- | --- |
| 2005 | Homozygosity for CNDP1 (CTG)_5_ is more common in the absence of DKD in patients with DM (type 1 and 2) | Caucasians, Arabics | Odds ratio 2.56 (95% CI 1.36–4.84) | 0.0028 | B. Janssen et al. [ 11] |
| 2007 | Homozygosity for CNDP1 (CTG)_5_ is more common in the absence of diabetic ESRD in patients with DM type 2 | European Americans | Odds ratio 1.65 (95% CI 1.00–2.54) | 0.02 | B. I. Freedman et al. [12] |
| 2008 | Homozygosity for CNDP1 (CTG)_5_ does not influence the risk of developing ESRD in patients with DM type 1 and proteinuria | Caucasians | Hazard ratio 0.79 (95% CI 0.44–1.39) | 0.41 | K. Wanic [24] |
| 2009 | Homozygosity for CNDP1 (CTG)_5_ is not more common in controls compared to patients with DM type 2 and ESRD | African Americans | - | 0.842 | C. W. McDonough et al. [23] |
| 2010 | Homozygosity for CNDP1 (CTG)_5_ is more common in the absence of DKD in women but not in men with DM type 2 | Caucasians | Odds ratio 0.5 (95% CI 0.30–0.68) | - | A. L. Mooyaart et al. [25] |
| 2010 | Homozygosity for CNDP1 (CTG)_5_ increases the risk for development of ESRD in patients with DM type 1 and DKD | Caucasians | Hazard ratio 2.19 (95% CI 1.21–4.01) | - | A. Alkhalaf et al. [26] |
| 2011 | No differences in CNDP1 (CTG)_n_ genotype frequencies between patients with DM type 2 with and without DKD | Scandinavians | - | 0.17 | T. S. Ahluwalia et al. [10] |
| 2015 | Homozygosity for CNDP1 (CTG)_5_ is associated with a higher risk of cardiovascular mortality in female patients with DM type 2 | Caucasians | Hazard ratio 1.77 (95 % CI 1.12–2.81) | - | A. Alkhalaf et al. [28] |
| 2016 | Homozygosity for CNDP1 (CTG)_5_ is more common in the absence of DKD in patients with DM type 2 | Indians | - | 0.035 | A. K. Yadav et al. [22] |
| 2017 | - Frequency of the homozygous CNDP1 (CTG)_5_ genotype increases with time on hemodialysis in patients with DM type 2 - Homozygosity for CNDP1 (CTG)_5_ is more common in the absence of biopsy-proven DKD in female patients with DM type 2 | Germans, Turks |  | <0.05 | T. Albrecht et al. [21] |
| 2019 | CNDP1 (CTG)_6_ is associated with a higher incidence of DKD in patients with DM type 2 in three ethnicities | Malaysians, Indians, Chinese | Odds ratio 2.46 (95% CI 1.48–4.10), 2.26 (95% CI 1.34–3.83), and 1.77 (95% CI 1.18–2.65) | - | M. J. Yahya et al. [9] |

Abbreviation: *DKD* diabetic kidney disease, *DM* diabetes mellitus, *ESRD* end-stage renal disease
